# The Effects of Relative Humidity on the Flowability and Dispersion Performance of Lactose Mixtures

**DOI:** 10.3390/ma10060592

**Published:** 2017-05-29

**Authors:** Xiang-Yun Lu, Lan Chen, Chuan-Yu Wu, Hak-Kim Chan, Tim Freeman

**Affiliations:** 1 School of Medical Instrument and Food Engineering, University of Shanghai for Science and Technology, Shanghai 200093, China; nephelelu@hotmail.com; 2 Freeman Technology Ltd., Tewkesbury GL20 8DN, UK; tim.freeman@freemantech.co.uk; 3 Deprtment of Chemical & Process Engineering, University of Surrey, Guildford GU2 7XH, UK; c.y.wu@surrey.ac.uk; 4 Advanced Drug Delivery Group, Faculty of Pharmacy, The University of Sydney, Sydney, NSW 2006, Australia; kim.chan@sydney.edu.au

**Keywords:** relative humidity, lactose, dry powder inhalation, flowability, dispersion

## Abstract

The flowability and dispersion behavior are two important physicochemical properties of pharmaceutical formulations for dry powder inhalers (DPIs). They are usually affected by the environmental conditions, such as temperature and relative humidity (RH). However, very few studies have been focused on the relationship between the two properties and their dependence on RH during storage. In this research, model pharmaceutical formulations were prepared using mixtures of coarse and fine lactose. The fractions of fines in the mixtures were 0%, 5%, 10%, and 20%, respectively. These blends were stored at four different RH levels, 0%, 30%, 58%, and 85%, for 48 h. The FT4 Powder Rheometer was used to evaluate the powder flowability, and the Malvern Spraytec^®^ laser diffraction system was employed to assess the powder dispersion performance. The results indicated that both the flow and dispersion properties of lactose blends deteriorate after being stored at 85% RH, but improved after being conditioned at 58% RH. The fine particle fractions (FPFs) of the blends with 5% and 10% fine fractions and the as-received coarse lactose decreased when they were conditioned at 30% RH. For the blend with 20% fine fraction, a high RH during storage (i.e., 85% RH) affected the dispersion property, but had a limited influence on its flowability, while, for the coarse lactose powder, the different RH conditions affected its flowability, but not the dispersion results. A strong correlation between the powder flowability and its dispersion performance was found.

## 1. Introduction

The flowability of powder materials is important for their handling and processing [1]. In the pharmaceutical industry, a good flowability of powders ensures dose accuracy and uniformity, and enables drug powders, such as dry powder inhalers (DPI), to be fluidised and released from the delivery system [2]. Several characterisation methods for the flow properties of bulk solids, e.g., the angle of repose, the determination of densities (for compressibility index and Hausner ratio), the mass flow rate, through orifice and shear cell tests, have often been used [3,4]. Usually, for cohesive powders, the flowability are often difficult to be differentiated by the methods above. In recent decades, the innovative dynamic flowability testing methods based on the FT4 Powder Rheometer were proved to not only correlate well with other measurement methods [5] but also be able to characterise the different fine powder flow properties [6].

The presence of free water during storage can dramatically change the powder flowability [7,8,9,10,11,12]. At the same time, micronised drug powders usually showed poor aerosol performance when they were exposed to an environment of high relative humidity (RH) [13,14,15,16]. However, the relationship between the flow and dispersion behaviors of powders is still not fully understood. Furthermore, it is still not clear if powder flowability measurements can be used to predict the dispersion behavior of powders for dry powder inhalation. Therefore, the aim of the present work was to explore the effects of RH during storage on powder flow properties and dispersion performance and to examine the correlation between flowability and dispersion performance of these blends.

## 2. Materials and Methods

Different inhalation grades of alpha-lactose monohydrate used in this study are Inhalac^®^ products with coarse lactose 120 (D_50_ = 132.0 μm) and fine lactose 400 (D_50_ = 8.4 μm) (Meggle Pharma, Wasserburg, Germany). They were thermodynamically stable [17]. The lactose blends with fine lactose fractions of 0, 5, 10 and 20 wt. % were prepared by sandwiching the fine lactose between the coarse one and mixing manually at a constant room of 22 ± 2 °C and an RH of 50% ± 10%.

### 2.1. Sample Preparation

The blends prepared as a thin layer in Petri dishes were conditioned at four RH values for 48 h, for which the RH was maintained in desiccators with dry powders and over-saturated salt solutions, including phosphorous pentoxide, calcium chloride, sodium bromide, and potassium chloride. The corresponding RH value in each container was 0%, 30%, 58%, and 85% at a temperature of 25 ± 1 °C. The RH and temperature were monitored over the whole conditioning period with a thermohygrometer (610 Testo AG, Lenzkirch, Germany). Caking phenomenon of powders was observed at high RH 85%.

### 2.2. Flow Property Measurement

**Figure 1:**
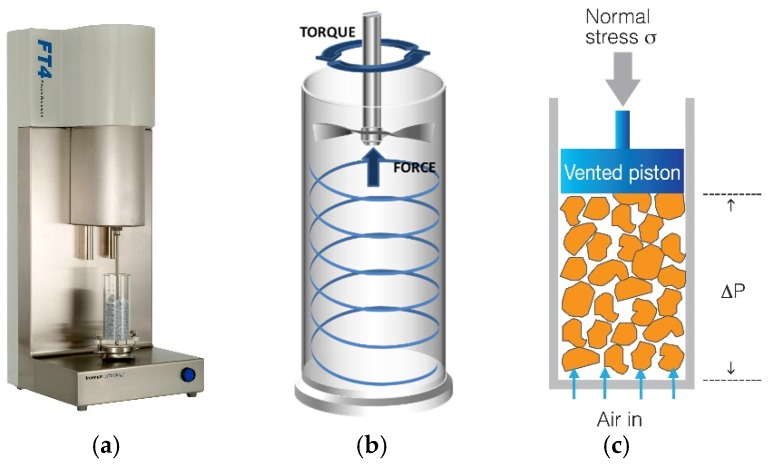
FT4 Powder Rheometer (**a**); flow energy measurement method (**b**) and permeability measurement method (**c**).

The standard dynamic test, permeability test, and shear cell test were performed to characterise the flowability of the lactose blends using the FT4 Powder Rheometer (Figure 1a). A conditioning cycle prior to any measurement was performed to remove the packing history and operator differences [18]. All measurements were repeated three times.

During the dynamic measurement, the blades was rotated and moved axially into the powder sample whilst the rotational torque and the axial force were measured as a function of the blade height within the powder bed as shown in Figure 1b. Thus, force measurements were converted into flow energy to determine the work done during the traverse, reflecting the resistance of powder to flow. The dynamic measurement consisted of seven identical repeated tests with a tip speed of 100 mm/s. Additionally, the variable flow rate tests were carried out at reducing blade tip speeds from 100, 70, 40, to 10 mm/s [5]. From these measurements, the following flow parameters were determined: (1) the normalised basic flow energy (BFE_Norm_), which is the energy required to move down the blade through one gram of powder; (2) the Flow Rate Index (FRI), a dimensionless parameter that is defined as the ratio between the flow energy of the powder at anticlockwise blade motions of 10 and 100 mm/s [6]; (3) the specific energy (SE), which is the energy required to move up the blade through one gram of powder in a clockwise motion at a 100 mm/s tip speed [18].

The permeability test was performed to evaluate the air resistance and/or ease of air permeation through the powder bed [19]. It measured the pressure drop across the powder bed whilst the applied normal pressure was varied as 1, 2, 4, 6, 8, 10, 12, and 15 kPa and the air velocity through the bed was maintained constant at 2 mm/s [5] (Figure 1c). The corresponding permeability *k* (cm^2^) can be calculated using simplified Darcy’s law as Equation (1) [20]:

(1) $$k=\frac{q\cdot \mu \cdot L}{\Delta P}$$

where *q* is the flux (cm/s), *μ* represents the air viscosity (1.74 × 10^−5^ Pa·s), *L* expresses the length of the powder bed (cm), and ∆*P* is the pressure drop across the powder bed (Pa).

A high air permeability is generally obtained for large particles as voids in the powder bed are large, reducing the pressure drop [21].

### 2.3. Dispersion Behaviors

It was shown that the laser diffraction technique could detect subtle differences between formulations and assess the dispersion performance that correlated well with measurement by inertial impaction [22]. In this paper, the laser diffraction method using Spraytec^®^ (Malvern Instruments Ltd., Worcestershire, UK) was employed as a fast-screening tool for analysis. The inhalation cell of a custom-made dry powder inhaler model (channel a) [23] was filled with the blends and inserted into the Spraytec. The airflow generated in the Spraytec^®^ was 60 L/min, and the focal length of the lens was 100 mm, which led to a measurable particle size range of 0.5–200 μm. The measurement was set to trigger when the laser transmission concentration was below 95%. The particulate refractive index used for these investigations was 1.533. The particle size distribution was calculated using Fraunhofer theory and the fine particle fraction (FPF) was then analysed from the Spraytec^®^ software based on the fraction of dispersed particles with a diameter smaller than 8.4 μm, which was the mean equivalent sphere volume diameter of the fine particle.

### 2.4. Analysis of Statistical Significance

Double-factor variance analysis was applied on BFE_Norm_, SE, and FRI of lactose blends with four fine fractions after being stored at four RH values. Univariate ANOVA with the generalised linear model was used to detect BFE_Norm_, SE, and ff_c_ at a level of significance of α = 0.05 by SPSS 19.0 (International Business Machines Corporation, New York, NY, USA).

## 3. Results

### 3.1. Effect of Relative Humidity on Dynamic Flow Properties

**Figure 2:**
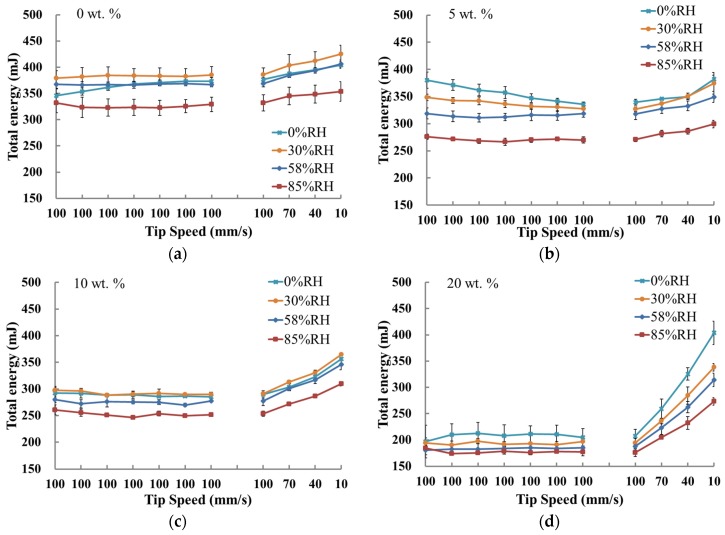
Flow energy for the treated samples with fine fractions (**a**) 0 wt. %; (**b**) 5 wt. %; (**c**) 10 wt. %; and (**d**) 20 wt. % at various relative humidities.

The good repeatability of all powders in the initial seven flow energy tests showed that all powders had a stable rheology [5]. It often assumes that, when considering the particle size, powders that flow freely under gravity result in higher flow energy, whereas cohesive powders show lower flow energy [5]. Therefore, the total flow energy shown in Figure 2a–d indicated that powders of higher fine fractions were more cohesive. Hence, the dynamic flow test was suitable for characterising the flowability of the lactose blends with different fine fractions.

However, for the blends with the same fine fraction, there was a rapid decrease in the flow energy at 85% RH due to the uptake of moisture (Figure 2a–d), so the flow properties became poorer. In addition, the flowability of the blend with a 20% fine fraction was very sensitive to the RH.

**Figure 3:**
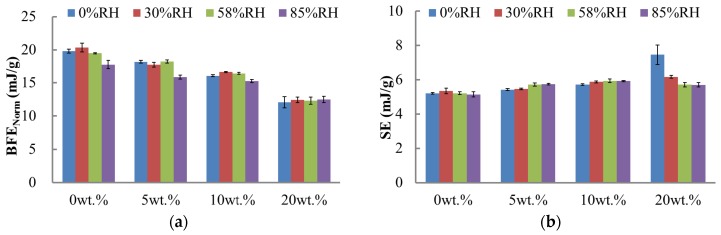

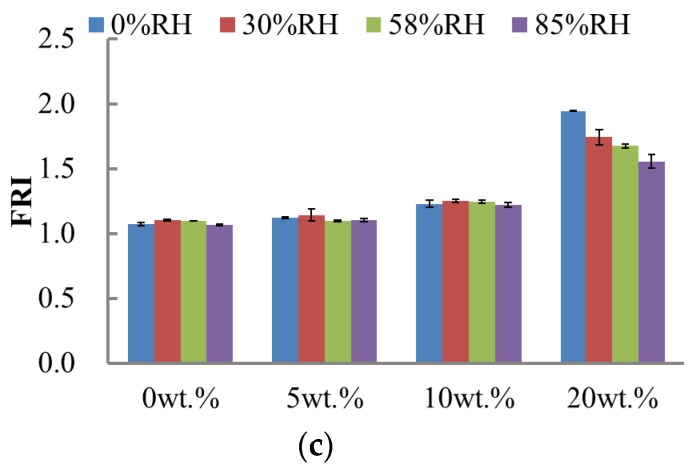
Flow properties obtained from the dynamic test with downward anti-clockwise and upward clockwise motion (error bars represent standard deviations, n = 3): (**a**) Normalised Basic Flow Energy (BFE_Norm_); (**b**) Specific Energy (SE); (**c**) Flow Rate Index (FRI).

The twisted blade method was adopted to measure the powder flow energy by downward and upward testing modes. These two movements showed bulldozing action along the entire blade length and shearing with minimal consolidation. Moreover, BFE_Norm_ and FRI were obtained from the former test mode and SE was measured by the latter. The double-factor variance analysis indicated that both the fine lactose fraction and RH influenced the BFE_Norm_, FRI, and SE. There was a significant difference in BFE_Norm_ values at the 20 wt. % fine fraction (*p* < 0.05) (Figure 3a). Significant differences were also observed in SE at 0% RH (*p* < 0.05) (Figure 3b). The fine lactose fraction of the blends was an important factor in dominating the flowability, in particular, at the highest lactose fraction (20 wt. %).

### 3.2. Effect of Relative Humidity on Permeability

**Figure 4:**
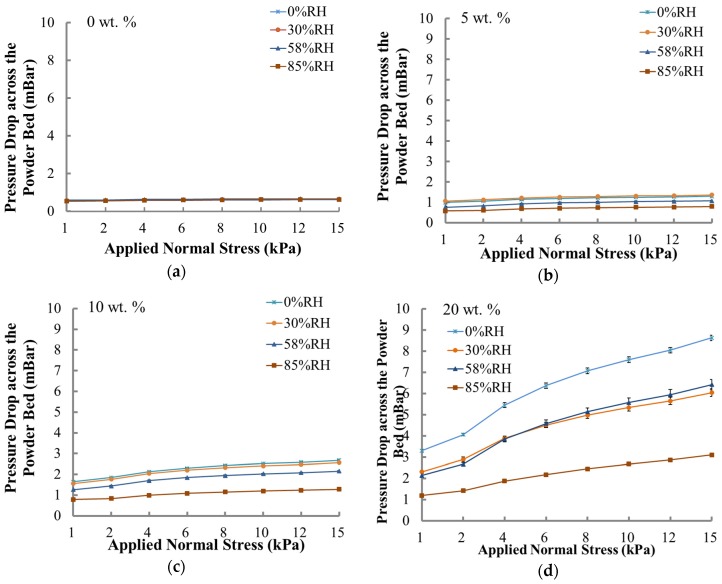
The relationship between the pressure drop across the powder bed and applied normal stress for blends with fine lactose fraction of (**a**) 0 wt. %; (**b**) 5 wt. %; (**c**) 10 wt. %; (**d**) 20 wt. %.

It can be seen from Figure 4a that the permeability of the coarse lactose was insensitive to all RH considered. As the fine lactose fraction increased (Figure 4b–d), the influence of the RH became more significant due to the increase in water uptake.

### 3.3. Effect of Relative Humidity on Dispersion Performance

**Figure 5:**
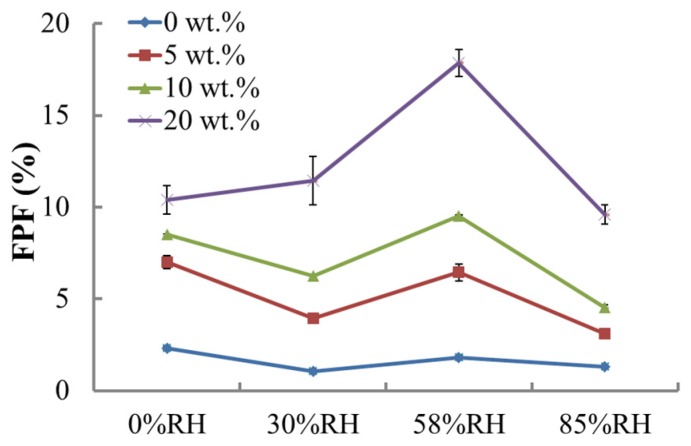
Changes in fine particle fractions (FPFs) (cut-off diameter equals to 8.4 μm) for blends of different fine fractions at various relative humidities.

Figure 5 showed that fine particle fractions (FPFs) for the pure coarse lactose (0%) at four different RH values were almost the same. For other blends considered, an RH of 58% seemed beneficial to the dispersion of lactose blends, especially for the blend with 20% fine lactose fraction. Furthermore, the FPFs of blends at 85% RH decreased dramatically.

## 4. Discussion

As the content of beta-lactose was extremely low and will be formed at above 93.5 °C [24], polymorphic transform would not happen at room temperature during these experiments. Thus, the impact of the conversion between the two forms was small and was ignored. At elevated RH, water molecules are likely to absorb onto the powder surface, and the interior moisture of the powders may exchange with the free water in the environment [25,26]. Above an RH of 65% in the air, capillary force, and Lifshitz-van der Waals force usually dominate the inter-particle force [27,28,29]. When the RH is below 65%, the inter-particle force is mainly dominated by the Lifshitz-van der Waals force and electrostatic force [27,30]. Since the capillary force (liquid bridge force) is dominated in powders at higher RH, the total energy values of the blends conditioned at 85% RH were much lower than the others, except for the 20 wt. % fine fraction powder (Figure 2). All the flowability parameters for the 20 wt. % blend were different from other blends of various fine fractions, such as BFE_Norm_, SE, and FRI in Figure 3. Figure 4 showed the powder permeability of the powder blends at different RH conditions. Obviously, the lower the fine fraction in the powders, the lower the pressure drop across the bed, whereas, due to the increasing inter-particular gaps and capillary voids within the powder bed [19], the 20 wt. % blend at an RH of 85% was more permeable (see Figure 4d). Overall, the fine lactose fraction is critical to the response of flowablity to the environmental conditions, since fine lactose is easier to absorb moisture [31].

Figure 5 showed that 58% RH is the optimal RH for the dispersion behavior, which to some extent is consistent with the observation reported in the literature [16]. The variations in the dispersion efficiency were dependent on the balance between the electrostatic force and the capillary force [16]. Wet agglomerates can exist when the powder is exposed to an RH of 85%, which led to the worst dispersion property of the lactose blends considered, which is in broad agreement with previous findings [14]. Moreover, the trends of FPFs with the storage RH was similar for all the lactose blends considered.

**Figure 6:**
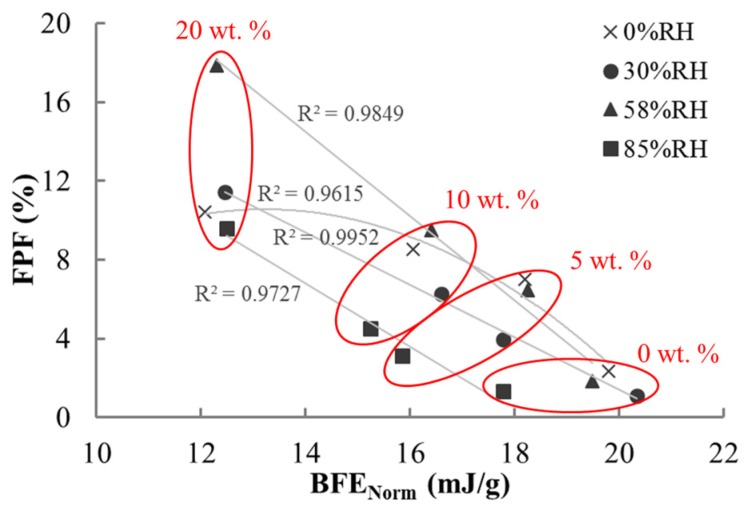
The relationship between FPF and BFE_Norm_ values of all the blends being treated at different RH levels.

Figure 6 showed the correlation between BFE_Norm_ and FPF for all cases considered in this study. As the fine lactose fraction increases, the FPF becomes more sensitive to the RH, while BFE_Norm_ becomes less sensitive to RH. This can be better illustrated with the ellipses superimposed in Figure 6, in which each ellipse covers all data points for a give blend. The major axis of the 4 ellipses changed from the vertical direction to the horizontal position, showing the different sensitivity to the RH. The flowability of lactose powder was relatively good at 30% and 58% RH levels. These results are in accordance with Lumay et al. [32]. At high humidity, the capillary force, namely the liquid bridge force, dominates the adhesion strength [27,28,29]. In terms of both the flow and dispersion properties of the coarse lactose, the 5 wt. % and 10 wt. % blends deteriorated after being conditioned at 85% RH due to the adverse effect of the liquid bridge force. Additionally, the relationships between FPF and BFE_Norm_ values were linear at 30, 58, and 85% RH. However, at 0% RH, the correlation followed a parabola due to the strong influence of electrostatic force on the fine particles. The knowledge of the adhesion forces between solid particles is essential to the powder flow [27] and dispersion [17]. The adhesive force is mainly due to the van der Waals force and capillary force at these RH conditions—except at 0% RH [27,28,29,30]. Considering the linear relationship between FPF and BFE_Norm_ at 30, 58, and 85% RH conditions, the electrostatic force must be the most significant factor for the non-linear phenomenon at 0% RH.

## 5. Conclusions

Dynamic flow tests and permeability measurement indicated that the fine lactose fraction, after being conditioning at different RH levels, is the dominating factor affecting the flowability of the lactose blends. The 5 wt. %, 10 wt. %, and 20 wt. % lactose blends had the worst flowability while being conditioned at 85% RH. Meanwhile, these blends showed the poorest dispersion performance. Nevertheless, the flowability of the 20 wt. % blend was insensitive to the effect of humidity storage. For all the lactose blends at an intermediate RH (say, 58%), the dispersion behaviors of powders were fairly good, implying that there is an optimal RH for promoting powder flow and dispersion in dry powder inhalers. In addition, it was found that there is a strong correlation between FPF and BFE_Norm_, so the measure of BFE_Norm_ could give a good indication of the dispersion performance of the blends in DPIs.
